# Shoes that restrict metatarsophalangeal dorsiflexion cause proximal joint compensations

**DOI:** 10.1186/1757-1146-5-S1-P28

**Published:** 2012-04-10

**Authors:** Dominic Thewlis, Gunther Paul, Chris Bishop

**Affiliations:** 1 School of Health Sciences, University of South Australia, Adelaide, South Australia, 5000, Australia; 2 Sansom Institute for Health Research, University of South Australia, Adelaide, South Australia, 5000, Australia; 3 Mawson Intitute, University of South Australia, Adelaide, South Australia, 5041, Australia

## Study aim

To describe barefoot, shod and in-shoe kinematics during stance phase of walking gait in a normal arched adult population.

## Materials and methods

An equal sample of males and females (n = 24) was recruited. In order to quantify the effect of footwear independent of technical design features, an ASICS shoe (Onitsuka Tiger-Mexico 66, Japan) was used in this study. Markers were applied to three conditions; barefoot, shod, and in-shoe. The calibration markers were used to define static *pose*. The order of testing was randomised. Participants completed five trials in each condition. Kinematic data were captured using a 12 camera VICON MX40 motion capture system at 100 Hz and processed in Visual3D. A previously developed model was used to describe joint angles [1]. A univariate two-way ANOVA was used to identify any differences between the pairs of conditions. Post-hoc Sheffé tests were used to further interrogate the data for differences.

## Results

**Figure 1:**
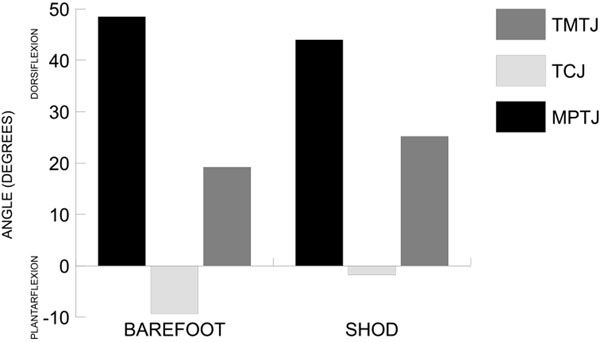
Joint angles (degrees) at peak hallux dorsiflexion

At peak hallux dorsiflexion (Figure 1), during propulsion, the metatarsophalangeal joint (MPTJ) was significantly more dorsiflexed in the barefoot condition compared to the shod condition (*p* = 0.004). At the same gait event, the tibiocalcaneal joint (TCJ) was significantly more plantarflexed than both the shod and in-shoe conditions (*p* < 0.001), and the tarsometatarsal joint (TMTJ) was significantly less dorsiflexed in the barefoot condition compared to the shod and in-shoe conditions (*p* < 0.001).

## Conclusions

The findings of the current study demonstrate that footwear has significant effects on sagittal plane MPTJ joint dorsiflexion at peak hallux dorsiflexion, which results in compensations at proximal foot joints.
